# 663. Incidence of Acute Kidney Injury (AKI) among Adult Hospitalized Patients (pts) with Carbapenem Resistant Gram-Negative Infections (CR-GNIs) Who Received Early Targeted Treatment (TX) with a Newer β-Lactam (BL)-, Colistin (COL)-, or Aminoglycoside (AG)-Containing Regimen

**DOI:** 10.1093/ofid/ofac492.715

**Published:** 2022-12-15

**Authors:** Thomas Lodise, Ryan J Dillon, Brian H Nathanson

**Affiliations:** Albany College of Pharmacy and Health Sciences, Albany, New York; Merck & Co., Inc, Rahway, New Jersey; OptiStatim LLC, Longmeadow, Massachusetts

## Abstract

**Background:**

Patients who develop AKI are at increased risk of adverse outcomes. The consequences of AKI are well-described in most therapeutic domains but there are scant data on the incidence of AKI among pts with CR-GNIs. This study sought to characterize the incidence of AKI and AKI-associated outcomes among adult, hospitalized pts with CR-GNIs who received early targeted tx with a newer BL, COL, or AG-containing regimen.

**Methods:**

Design: multi-centered observational study using 2016-20 data from the PINC AI Healthcare Database. Inclusion criteria: hospitalized; age ≥ 18 yrs; diagnosis for pneumonia, complicated urinary tract infection (cUTI), or bloodstream infection; CR-GN on culture site consistent with diagnosis; receipt of a newer BL, COL, or AG within 3 days of index CR-GN culture; and ≥3 consecutive days of a newer BL, COL, or AG (first received for ≥3 d defined tx group). Exclusion criteria: receipt of any renal replacement therapy prior to index tx day; and serum creatinine (S_CR_) ≥2 mg/dL ± 2 d of index tx initiation day. Outcomes: on-tx AKI based on RIFLE criteria (50% increase in tx day 1 S_CR,_ assessed from tx day 2 through 3 days after BL, COL, or AG discontinuation); in-hospital mortality, and hospital LOS post-index culture day. Incidence of AKI was compared between tx groups and logistic regression (LR) was used to adjust for baseline differences. In-hospital mortality and hospital LOS post-index culture were compared between AKI groups.

**Results:**

Overall, 1,061 pts met criteria and the majority received BL (45%), followed by AG (36%), and COL (19%). We present baseline characteristics by treatment in **Table 1**. The rate of AKI was highest among COL (COL: 27.1% vs BL: 13.5% vs AG: 16.3%, p< 0.01). In the LR (**Figure 1**), receipt of COL was associated with a higher adjusted probability of AKI relative to BL and AG (p< 0.01). Occurrence of AKI was associated with higher mortality (AKI: 19.3% vs. No AKI: 5.9%, p< 0.01) and increased median [IQR] post-index culture LOS (AKI: 15 d [9-29] vs No AKI: 9 d [6-15], p< 0.01).

**Figure:**
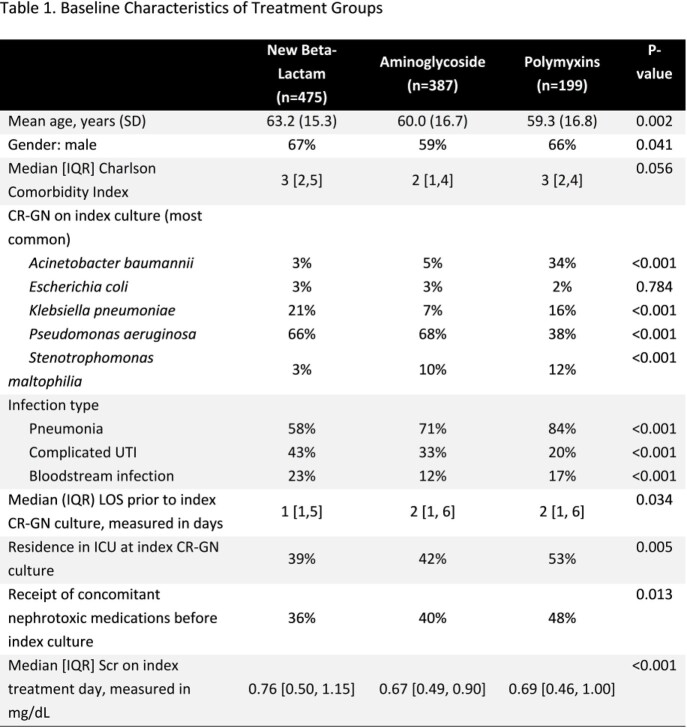

**Figure:**
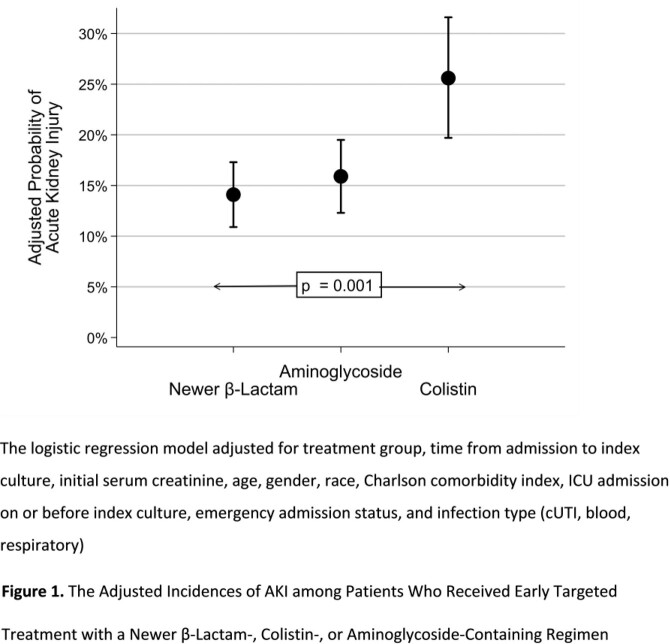

**Conclusion:**

Incidence of AKI among hospitalized pts with serious CR-GNIs was highest in pts who received an early-targeted COL-containing regimen. Occurrence of AKI was associated with increased in-hospital mortality and longer post-index culture hospital LOS.

**Disclosures:**

**Thomas Lodise, Jr., Pharm.D., PhD**, BioFire Diagnostics: Grant/Research Support|cidara: Advisor/Consultant|cidara: Honoraria|Entasis: Grant/Research Support|Merck: Advisor/Consultant|Merck: Grant/Research Support|Paratek: Advisor/Consultant|Shionogi: Advisor/Consultant|Spero: Advisor/Consultant|Venatrox: Advisor/Consultant **Ryan J. Dillon, MSc**, Merck: Employee|Merck: Stocks/Bonds **Brian H. Nathanson, Ph.D.**, cidara: Grant/Research Support|Merck: Advisor/Consultant|Merck: Grant/Research Support.

